# Non-Coding RNAs: Emerging Therapeutic Targets in Spinal Cord Ischemia–Reperfusion Injury

**DOI:** 10.3389/fneur.2021.680210

**Published:** 2021-09-08

**Authors:** Xiao Ling, Jun Lu, Jun Yang, Hanjun Qin, Xingqi Zhao, Pengyu Zhou, Shaoyi Zheng, Peng Zhu

**Affiliations:** ^1^Department of Cardiovascular Surgery, Nanfang Hospital, Southern Medical University, Guangzhou, China; ^2^Department of Orthopaedics, Nanfang Hospital, Southern Medical University, Guangzhou, China

**Keywords:** non-coding RNAs, spinal cord ischemia-reperfusion injury, microRNA, long non-coding RNA, cerebral ischemic diseases

## Abstract

Paralysis or paraplegia caused by transient or permanent spinal cord ischemia–reperfusion injury (SCIRI) remains one of the most devastating post-operative complications after thoracoabdominal aortic surgery, even though perioperative strategies and surgical techniques continue to improve. Uncovering the molecular and cellular pathophysiological processes in SCIRI has become a top priority. Recently, the expression, function, and mechanism of non-coding RNAs (ncRNAs) in various diseases have drawn wide attention. Non-coding RNAs contain a variety of biological functions but do not code for proteins. Previous studies have shown that ncRNAs play a critical role in SCIRI. However, the character of ncRNAs in attenuating SCIRI has not been systematically summarized. This review article will be the first time to assemble the knowledge of ncRNAs regulating apoptosis, inflammation, autophagy, and oxidative stress to attenuate SCIRI. A better understanding of the functional significance of ncRNAs following SCIRI could help us to identify novel therapeutic targets and develop potential therapeutic strategies. All the current research about the function of nRNAs in SCIRI will be summarized one by one in this review.

## Introduction

Up to 45% of the reported cases of spinal cord ischemia–reperfusion injury (SCIRI) were attributed to an iatrogenic cause (1). SCIRI remains one of the major concerns for aortic surgery as a result of postoperative paraplegia, with the overall incidences of 0.5–1.5% for coarctation repair, 10% for thoracic aneurysm repair, and around 20% for thoracoabdominal aorta repair (2). Despite the dramatic progress in surgical techniques and perioperative strategies, SCIRI, which can cause some extremely devastating postoperative complications, including paralysis and paraplegia, still cannot be prevented completely. Furthermore, the incidence of postoperative neurological deficit and permanent paraplegia induced by SCIRI remains as high as 9–16% (3, 4) and 0.3–6.5%, respectively (5).

Although occurring less frequently than cerebral ischemic diseases, SCIRI results in high mortality and disability rate and reduced quality of life. In contrast to cerebral ischemic diseases, which have well-established guidelines, no consensus guideline exists for the treatment of spinal cord ischemia (6). At present, lumbar cerebrospinal fluid drainage and epidural hypothermia protocols have been widely accepted as preventative measures for SCIRI (7). Monitoring of somatosensory and motor-evoked potential during the operation has also been demonstrated to help reduce the incidence of ischemic events (8). Moreover, several studies verify that those patients whose intra-operative blood pressure was maintained above 55 mmHg had a lower rate of neurologic deficits (9, 10). Unfortunately, there are scarce clinical management experience and data of spinal cord ischemia until now. Therefore, it is urgently necessary to develop definitive and effective therapeutic approaches for SCIRI.

Since the post-genome sequencing era, there is increasing evidence to prove that non-protein-coding regions account for more than 98% of the human genome, 80% of which are transcribed into RNAs (11). For a long time, the non-coding RNAs (ncRNAs) are mistaken for the “noise” of transcription. Recently, the expression, function, and mechanism of ncRNAs have attracted wide attention. To develop more effective and promising therapeutic agents for treating SCIRI, we need to have a better understanding of how various ncRNAs mediate the pathophysiological mechanisms of SCIRI.

## The Classification of Non-Coding RNAs

**Figure 1:**
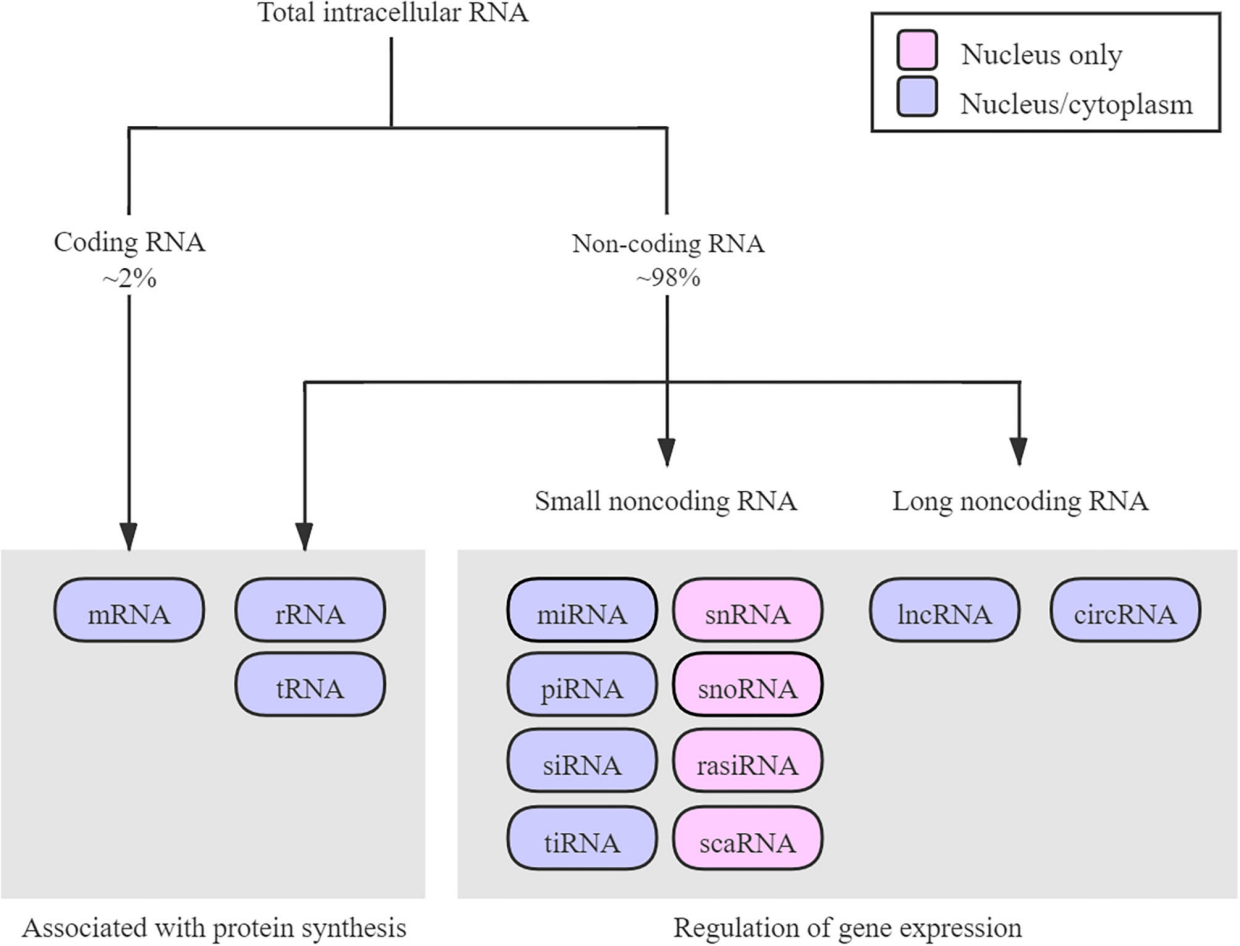
Diagram about the RNA family. According to molecular size, shape, function, and localization, ncRNAs are divided into different types. Most of the RNAs exert regulatory functions. Only ~2% of RNAs are coding protein. circRNA, circular RNA; lncRNA, long non-coding RNA; mRNA, messenger RNA; miRNA, microRNA; piRNA, piwi-interacting RNA; rRNA, ribosome RNA; rasiRNA, repeat-associated small interfering RNA; scaRNA, small cajal body-specific RNA; siRNA, small interfering RNA; snRNA, small nuclear RNA; snoRNA, small nucleolar RNA; tRNA, transfer RNA; tiRNA, transfer RNA-derived stress-induced small RNA.

ncRNAs are functional molecules that originate in the genome but cannot be translated to proteins. According to molecular size, shape, and function, ncRNAs are divided into different types (Figure 1), including ribosomal RNAs (rRNAs), transfer RNAs (tRNAs), short interfering RNAs (siRNAs), piwi-interacting RNAs (piRNAs), microRNAs (miRNAs), circular RNAs (circRNAs), and long non-coding RNAs (lncRNAs) (12, 13).

Although rRNAs and tRNAs have been introduced as translation-related RNAs for several decades, other ncRNAs were previously regarded as transcriptional “murmur,” owing to their unclear physiologic functions. However, recent studies have shown that ncRNAs are a product of pervasive transcription (13, 14) and perform many biological functions, such as transcriptional and translational regulation, epigenetic modifications, and protein and RNA scaffolding, thereby adding a new layer of genomic regulation (15–18). Therefore, a more in-depth study of ncRNAs could help us to better understand the pathophysiology of humans (19).

miRNAs are a class of highly conserved non-coding RNAs with a length of about 17 to 22 nucleotides. miRNAs can bind to the 3′-untranslated region (3′-UTR) of target messenger RNA (mRNA) through complementary base pairing. This interaction allows the inhibition of their target mRNA and the negative regulation of gene expression after transcription (20). Each miRNA can bind to various target genes, while one same gene can also be conducted with several miRNAs (21). It has been demonstrated that miRNAs are ubiquitous in the human body, which have been associated with inflammation, tumor, and various diseases (22).

piRNAs are regarded as another large class of ncRNAs, with a length of 24 to 31 nucleotides and a lack of sequence conservation between species (23). Although their functional roles and method of biogenesis remain poorly understood, piRNAs can maintain genome integrity and prevent mutations through controlling the post-transcriptional silencing of retrotransposons (24).

lncRNAs are another type of RNA with a length longer than 200 nucleotides. So far, more than 3,000 lncRNAs have been identified, of which one-third are involved in various biological processes, such as cell growth, differentiation, apoptosis, and proliferation (25–28). The mechanism of lncRNA regulation at the transcriptional and post-transcriptional levels is, respectively, through the regulation and modification of chromosomes, resulting in changes in gene expression, and participation in RNA degradation as a source of competition for endogenous RNA and miRNA. Given their display of a wide variety of biological functions, lncRNAs have recently become a research hotspot (29).

circRNAs are a subclass of lncRNAs which have a covalently closed cyclic structure and lack a polyadenylated tail. Compared with linear RNA, circRNAs are more stable and resistant to RNA exonuclease by reason of their covalently closed loop structure (30). Recent studies have suggested that circRNAs are involved in regulating gene expression by acting as an RNA binding sponge, miRNA protein sponge, and translational regulator (31, 32). Therefore, circRNAs have great potential to become another promising and effective therapeutic target.

## Non-Coding RNAs in Spinal Cord Ischemia–Reperfusion Injury

SCIRI usually occurs during thoracoabdominal aortic surgery and cardiac surgery, which can cause some unpredictable and extremely devastating complications, including paralysis and paraplegia. Two distinct phases of SCIRI influence the occurrence of disability. The first phase is ischemia, which occurs during a period of circulatory arrest or aortic cross-clamping. The second one is reperfusion, which results in intracellular calcium overload, the release of oxygen-free radicals, inflammatory response, and neuronal apoptosis (33, 34).

**Figure 2:**
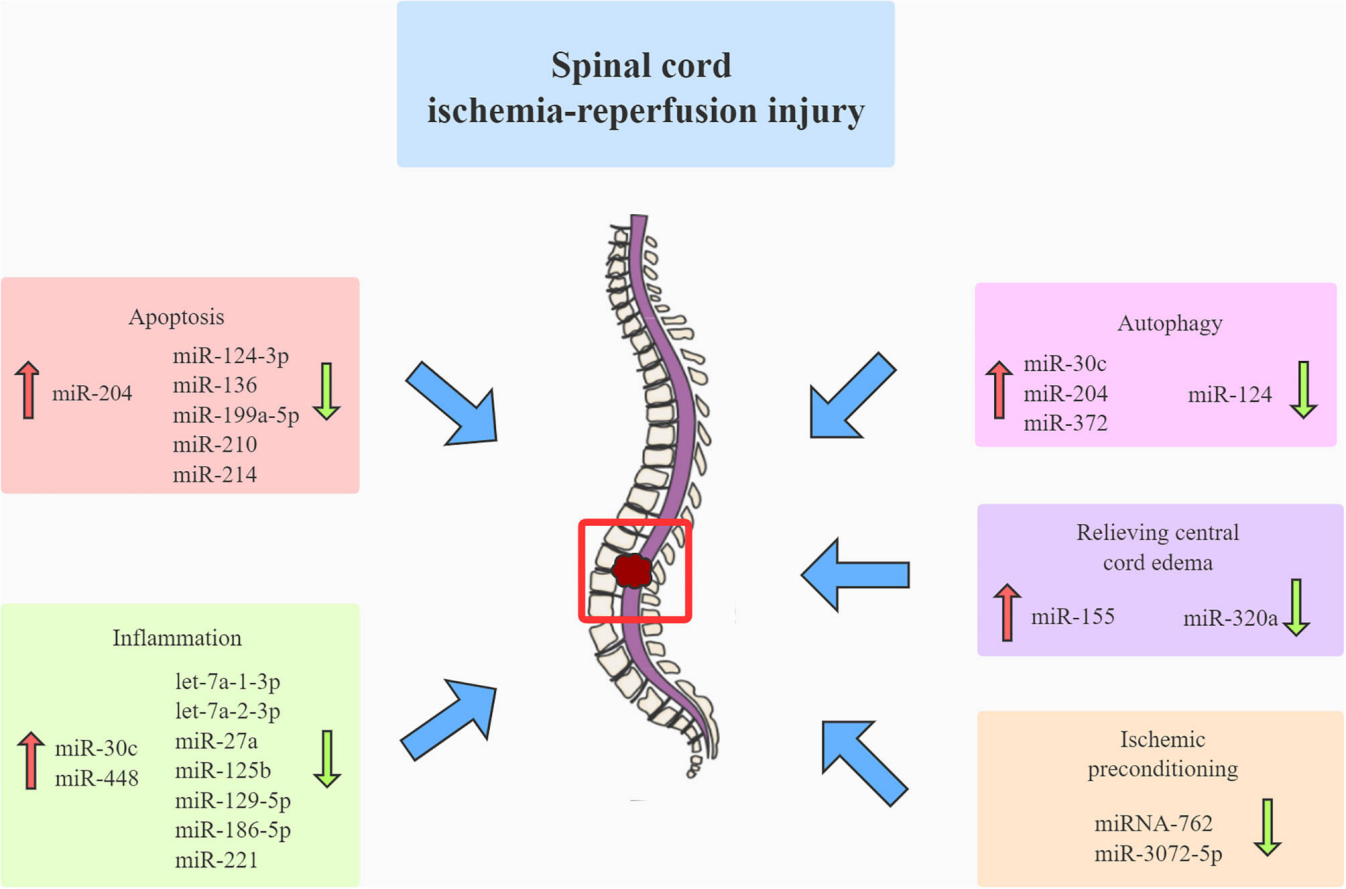
Many miRNAs contribute to the pathophysiology of spinal cord ischemia-reperfusion injury. Mounting evidence has suggested that various miRNAs contribute to the pathophysiology of spinal cord ischemia-reperfusion injury by mediating apoptosis, autophagy, inflammation, relieving central cord edema, and ischemic preconditioning. miRNA, microRNA.

In contrast to cerebral ischemic diseases, which have well-established guidelines, no consensus guideline exists for the treatment of SCIRI (6). The current strategies in our clinical setting (including hypothermia, distal aortic perfusion, and cerebrospinal fluid drainage) fail to effectively mediate spinal cord protection against ischemia–reperfusion injury (IRI) due to their inherent limitation (35). Therefore, a more effective strategy should be developed to attenuate SCIRI. Mounting evidence suggested that many ncRNAs played critical roles in the pathophysiology of SCIRI (Figure 2). Hence, to design more innovative and effective therapeutic agents for treating SCIRI, we should better understand the function of various ncRNAs mediating the pathophysiological mechanisms underlying SCIRI.

### MicroRNAs in Spinal Cord Ischemia–Reperfusion Injury

Considered as a major breakthrough, miRNAs are regarded as therapeutic targets for SCIRI for at least two reasons: (1) a single miRNA can regulate various target genes and affect the whole gene network and (2) miRNAs can be efficiently targeted by several tools to elevate the levels of miRNAs with beneficial functions or reduce the levels of pathogenic miRNAs (36) (Table 1).

**Table 1:** The expression, mechanism, and function of a broad array of miRNAs in SCIRI.

**miRNA**	**Animal or/and cells**	**Model**	**Expression**	**Target/mechanisms**	**Function**	**References**
let-7a-1/2-3p	C57BL/6 mice Microglia cells	SCIRI OGD/R	**↓**	Downregulate HMGB1	Inhibit microglia activation	(37)
miR-21	Wistar rats	SCIRI	No significant differences	Target and negatively regulate Faslg and PDCD4	Anti-apoptosis	(38)
miR-21	Female C57BL/6 mice Primary astrocytes	SCIRI OGD/R	**↓**	Target Gpc6 and GDNF through increasing the level of STAT3	Promote the transformation of A1s into A2s, synapse formation, and neurite growth	(39)
miR-22-3p	SD rats	SCIRI	**↓**	Negatively regulate IRF5	Inhibited inflammation	(40)
miR-22-3p	SD rats	SCIRI	**↑**	Possibly regulate MAPK signaling		(41)
miR-25	SD rats	SCIRI	No significant differences	Inhibit NADPH oxidase 4 expression	Attenuate the oxidative stress	(42)
miR-27a	SD rats	SCIRI	**↓**	Downregulate TICAM-2 of TLR_4_ signaling	Ameliorate BSCB inflammatory	(43)
miR-30c	SD rats PC12 cells	SCIRI OGD/R	**↑**	Target and negatively regulate SIRT1 level	Inhibit autophagy, promote apoptosis and inflammation	(44)
miR-30c	SD rats SY-SH-5Y cells	SCIRI OGD/R	**↑**	Negatively regulate Beclin-1 and LC3II expression	Inhibit autophagy, promote apoptosis	(45)
miR-124	Wistar rats	SCIRI	**↓**	Negatively regulate the expressions of iA-SPP, becline-1, and LC3-II	Inhibit mitophagy, promote apoptosis	(46)
miR-124-3p	SD rats	SCIRI	**↓**	Target and negatively regulate Ern1 and enhance M2 macrophages polarization	Impeded cell apoptosis and attenuated tissue impairment and nerve injury	(47)
miR-125b	SD rats VSC4.1 motor neuron	SCIRI OGD/R	**↓**	Prevent the activation of p53 network through targeting the decreasing TP53INP-1	Inhibit neuroinflammation and attenuated neuronal apoptosis	(48)
miR-129-5p	C57BL6 mice	SCIRI	**↓**	Inhibit HMGB1 and the TLR_3_-cytokine pathway	Ameliorate neuroinflammation and BSB damage	(49)
miR-136	SD rats AGE1.HN and PC12 cells	SCIRI OGD/R	**↓**	Target and inhibit TIMP3	Attenuate apoptosis	(50)
miR-155	C57Bl/6 mice Endothelial cells, MN-1 cells	SCIRI LPS	**↑**	Target and negatively regulate Mfsd2a	Aggravate central cord edema and des-troy blood–brain barrier integrity	(51)
miR-186-5p	SD rats	SCIRI	**↓**	Attenuate the upregulation of Wnt5a, TLR3, and CXCL13	Inhibit neuroinflammation	(52)
miR-199a-5p	SD rats	SCIRI	↓	Downregulate the expressions of ECE1	Anti-apoptosis	(11)
miR-204	AGE1.HN and PC12 cells	Hypoxic	↑	Inhibit Bcl-2	Promote apoptosis	(53)
miR-204	C57/BL6 mice AGE1.HN and PC12 cells	SCIRI Hypoxic	↑	Inhibit RAP2C	Promote apoptosis	(54)
miR-204	Wistar rats	SCIRI	↑	Downregulate the expressions of Bcl-2, the LC3-II/LC3-I ratio, and beclin-1	Inhibit autophagy and promote apoptosis	(55)
miR-214	Male SD rats PC12 nerve cells	SCIRI OGD/R	↓	Target and negatively regulate Kcnk2	Promote apoptosis	(56)
miR-221	AGE1.HN and PC12 cells	OGD/R	↓	Target and negatively regulate TNFAIP2	Alleviate the inflammatory response and cell apoptosis	(57)
miR-320	Wistar rats	SCIRI	No significant differences	Negatively regulate the expression of phospho-Hsp20	Promote apoptosis	(58)
miR-320a	SD rats	SCIRI	↓	Targeting downregulated AQP1 expression	Reduce spinal cord edema	(59)
miR-372	SD rats PC12 cells	SCIRI Hypoxia	↑	Target and negatively regulate Beclin-1	Inhibit autophagy	(60)
miR-448	SD rats AGE1.HN and PC12 cells	SCIRI OGD/R	↑	Downregulate SIRT1	Promote apoptosis	(61)
miR-485-5p	AGE1.HN and SY-SH-5Y cell	TNF-α induced	Unknown	Targeting suppressed TRADD expression	Anti-apoptosis	(62)
miR-762 and miR-3072-5p	Male C57BL/6 mice	SCIRI	↓	Increase plasma VEGF level	Protect against organ ischemia	(63)

*↑ and ↓ indicate the increase and decrease expression respectively*.

*Bcl2, B-cell lymphoma-2; ECE1, endothelin-converting enzyme 1; Ern1, endoplasmic reticulum to nucleus signaling 1; HMGB1, high-mobility group box-1; iASPP, inhibitory member of the apoptosis-stimulating proteins of p53 family; Mfsd2a, major facilitator superfamily domain containing 2a; OGD/R, oxygen-glucose deprivation/reoxygenation; PDCD4, programmed cell death 4; SCIRI, spinal cord ischemia-reperfusion injury; SD rats, Sprague-Dawley rats; SIRT1, sirtuin 1; TLR3, Toll-like receptor-3; TLR4, Toll-like receptor-4*.

#### Apoptosis

Apoptosis can lead to tissue loss in ischemic lesions. Various research have confirmed the character of miRNAs in ameliorating neuronal apoptosis after spinal cord ischemia. He and his colleagues reported that the overexpression of miRNA-21 exerted neuroprotective effects in a rodent model of SCIRI, possibly *via* the downregulated expressions of Fas ligand, programmed cell death 4, and caspase-3 (38). Su and his team demonstrated that naive astrocytes were transformed into A2s *in vivo* by ischemia. The *in vitro* silencing of miR-21 can transform neurotoxic reactive astrocyte A1 into A2 and promote synapsis formation and nerites growth by targeting Gpc6 and GDNF through the STAT3 signaling pathway after oxygen-glucose deprivation (OGD) (39). Li and his co-workers showed that miRNA-124-3p in exosomes derived from bone marrow mesenchymal stem cell ameliorates nerve injury and tissue impairment and impedes cell apoptosis in a SCIRI rat model by reducing the expression of Ern1 and augmenting the polarization of M2 macrophages (47).

Another study found that, in SCIRI rats and hypoxic nerve cells, miR-136 decreased, and tissue inhibitor of metalloproteinase-3 (TIMP3) increased. Cells (AGE1.NH and PC12 cell lines) transfected with miR-136 mimic protected neurocytes against hypoxia, while cells co-transfected with pcDNA- TIMP3 abrogated the results induced by the overexpression of miR-136 (50). miR-199a-5p was also identified as apoptosis-related miRNA, which decreased in the rat spinal cord following IRI and targeted endothelin-converting enzyme 1 (ECE1). Upregulating miR-199a-5p might protect the spinal cord against IRI by negatively regulating ECE1 (11).

B-cell lymphoma-2 (Bcl2), a well-known anti-apoptotic protein, was confirmed to be negatively targeted and regulated by miR-204 in neuronal cells exposed to hypoxia. In the neuronal lines (AGE1.HN and PC12) induced by hypoxia, the expression of Bcl2 in neurocytes was lower than in the control group, while miR-204 expression was higher. Bcl-2 expression was enhanced by miR-204 inhibitor and inhibited by miR-204 mimic (53). A similar study showed that hypoxia significantly increased the apoptosis rate and downregulated the expression of RAP2C. Moreover, miR-204 could bind to the 3′-UTR of RAP2C. miR-204 inhibitors elevated the activity and expression of RAP2C in the neuronal lines (AGE1.HN and PC12) subjected to hypoxia, while miR-204 mimics downregulated the activity and expression of RAP2C. The miR-204 inhibitor and the overexpression of RAP2C could attenuate apoptosis in the cells treated with hypoxia (54).

Foxd3 upregulated miR-214 which can inhibit the expression of Kcnk2, thus resulting in reduced viability and increased apoptosis of PC12 cells under hypoxia/reoxygenation conditions. However, the overexpression of Kcnk2 or knockdown of Foxd3 enhanced the cell viability and inhibited the apoptosis of the hypoxia/reoxygenation-treated PC12 cells (56).

Hsp20 is a member of the small heat-shock protein family performing many physiologic functions, including anti-apoptosis effects (64–66), the regulation of protein aggregation, and the maintenance of the cellular cytoskeleton. Inhibiting miR-320 could protect the spinal cord against IRI *in vivo* by dramatically upregulating the expression of phospho-Hsp20 (58).

#### Inflammation

Recent studies have demonstrated that inflammation involves the pathological mechanism of IRI (67, 68). Proinflammatory cytokines have been implicated in damage to blood–spinal cord barrier (BSCB) integrity (69, 70). The BSCB consists of continuous capillary endothelial cells, surrounded by astrocytes, pericytes as well as perivascular microglia (71). During SCIRI, the microglia are widely introduced around the injury site as the primary responding immune cell. The activated microglia can aggravate ischemic nerve damage *via* activating phagocytes or secreting inflammatory cytokines (72).

In the OGD-stimulated microglial cells, Na et al. found that dexmedetomidine (DEX) led to the inactivation of microglial cells by upregulating the let-7a-1-3p and let-7a-2-3p expression. High-mobility group box-1 (HMGB1) was targeted and negatively mediated by let-7a-1-3p and let-7a-2-3p. An intraperitoneal injection of DEX improved the motion function of mice in a SCIRI model, mediated by the let-7a-1/2-3p/HMGB1 pathway (37).

Recently, miR-22-3p has been found to be downregulated in rat models of SCIRI. Increased miR-22-3p can facilitate the M2 polarization of macrophages, relieve inflammation, and reduce the degree of severity of SCIRI by repressing IRF5 (40). In contrast, another study found that miR-22-3p was persistently overexpressed in both 24- and 48-h groups by bioinformatics analysis and qRT-PCT (41).

An intrathecal infusion of the miR-27a mimics attenuated inflammatory damage to the BSCB in a rat model after SCIRI by negatively regulating the Toll-like receptor adaptor molecule 2 of the Toll-like receptor 4 (TLR4) signaling pathway and inhibiting the NF-κB/IL-1β pathway, whereas pretreatment with miR-27a anti-miR oligonucleotide significantly aggravated the inflammatory injuries (43).

Sirtuin 1 (SIRT1) acts as an essential role in inflammation submitted to IRI (73). Wang et al. showed that the depletion of miR-30c protected against inflammation and cell apoptosis by increasing the SIRT1 level in OGD-treated PC12 cells and SCIRI-treated rats (44). Another study found that the miR-125b expression was markedly downregulated at 12 h after SCIRI. Treatment with miR-125b mimic before ischemia decreased the expression of tumor protein 53-induced nuclear protein 1, p53, cytokines IL-1β, TNF-α, and cleaved caspase 3, thereby protecting the neurons against neuroinflammation, whereas the miR-125b inhibitor did not have the effects as mentioned above (48). Moreover, pretreatment with miR-129-5p mimic by intrathecal administration protected the neurons and BSCB against inflammatory responses after transient SCIRI by downregulating HMGB1 expression and inhibiting Toll-like receptor-3 (TLR3)-cytokine pathway activation, but rHMGB1 and TLR3 agonist Poly (I:C) reversed these effects (49).

miR-186-5p was found to be downregulated in the SCIRI rat model. Pre-treatment with the miR-186-5p mimic improved the neurological function, attenuated BSCB leakage and spinal cord edema, and decreased IL-15, IL-6, IL-1β, TNF-α, Wnt5a, TLR3, and chemokine ligand 13 expressions (52).

Intriguingly, in OGD-AGE1.HN and SY-SH-5Y cells, the inflammation and cell apoptosis were enhanced by miR-221 knockdown and attenuated by miR-221 overexpression *via* negatively regulating TNF-α induced protein 2 (57). Chen et al. found that the serum TNF-α expression was significantly elevated in patients who suffered from SCIRI. TNF-α treatment caused cell apoptosis *in vitro* (AGE1.HN and SY-SH-5Y), whereas this effect was reversed by hydrogen sulfide (H2S). H2S suppressed tumor necrosis factor receptor type 1-associated DEATH domain protein expression via promoted miR-485-5p expression (62). miR-448 was another miRNA reported as mediating SIRT1 to improve neurological function and inhibit the apoptosis of nerve cells. Downregulated miR-448 could contribute to relieving SCIRI through upregulating SIRT1 in SCIRI tissue and hypoxia-treated cells (61).

#### Autophagy

Autophagy, as the name implies, is that process wherein autophagosomes deliver the cargo they engulfed in the cytoplasm to lysosomes (74, 75). Autophagosomes can engulf cellular material in bulk and remove damaged organelles and unwanted cytosolic proteins (76).

Li and his team found that miR-30c downregulated the expression of Beclin-1 in SY-SH-5Y cells subjected to OGD, and H_2_S protected the spinal cord and induced autophagy *via* downregulating miR-30c expression and upregulating Beclin-1 and LC3II expression in the rat model of SCIRI. However, the administration of 3-methyladenine (3-MA), an inhibitor for autophagy, could reverse the spinal cord-protective effect of H_2_S (45). Yan et al. found that miR-204 was also an autophagy-related gene. The transient ischemia of spinal cords enhanced the ratio of LC3-II/LC3-I and reduced Bcl-2. AntagomiR-204 significantly reduced the level of caspase-3 and dramatically upregulated the expressions of Beclin-1, LC3-II/ LC3-I ratio, and Bcl-2 in spinal cords after reperfusion. 3-MA diminished the protective effect on the hind-limb motor function of antagomiR-204 (55). The miR-372 is another autophagy-related miRNA. Interference of this particular miRNA could reduce nerve cell apoptosis in SCIRI *via* increasing Beclin-1-mediated autophagy (60).

Recently, it has been found that mitophagy is a selective autophagic process for the mitochondria. In response to metabolic demand, mitophagy can regulate mitochondrial numbers and eliminate damaged mitochondria (77). In a SCIRI rat model, treatment with antagomiR-124 enhanced the expressions of mitochondrial Becline-1, LC3-II, and inhibitory member of the apoptosis-stimulating proteins of the p53 family in spinal cords. Inhibiting miRNA-124 may exert neuroprotection on the spinal cord by inducing mitophagy and anti-apoptotic effects. However, the administration of 3-MA could abrogate the neuroprotective effects of antagomiR-124 (46).

#### Reactive Oxygen Species Oxidative Stress

Reactive oxygen species (ROS) are another major mediator of the neurologic injury submitted to SCIRI. The sole purpose of NADPH oxidase (NOX) is to produce ROS (78). To date, there are seven known NOX isoforms, of which NOX4 is strongly associated with cerebral ischemia. Because of the organ-specific effect of NOX4 in neurons and blood–brain barrier endothelial cells, NOX4 is highly sensitive to cerebral ischemic damage (79).

Zhao and his colleagues found that pretreatment with miRNA-25-enriched exosomes by intrathecal injection diminished NOX4 expression in SCIRI rats, elevated superoxide dismutase activity, and decreased the levels of TNF-α, IL-1b, and malondialdehyde content in spinal cords, thus giving better neuroprotective effects (42).

#### Ischemic Preconditioning

Ischemic preconditioning (IPC) has protective effects against IRI in different organs. However, the mechanisms underlying the protective effects of IPC are still poorly understood. Recently, Ueno et al. demonstrated that miRNA-762 and miR-3072-5p were downregulated in the rat after remote IPC compared with those in sham-operated control and probably bound to the 3′-UTR of vascular endothelial growth factor (VEGF) mRNA. Anti-miR-762 and anti-miR-3072-5p can induce CD34-positive BM cells to produce a higher level of plasma VEGF to protect spinal cord neurons against ischemia (63).

#### Relieving Central Cord Edema

The integrity of BSCB is crucial to the spinal cord. On the contrary, the breakdown of BSCB allows circulating immune cells and exogenous pathogens to infiltrate the central nervous system, which can lead to disastrous consequences, such as central sensitization, neuronal loss, and glial remodeling (80, 81).

Major facilitator superfamily domain-containing 2a (Mfsd2a) is implicated in the establishment of a functional blood–brain barrier and the maintenance of its integrity, and Mfsd2a^−/−^ mice present with blood–brain barrier leakage due to increased transcellular trafficking across the endothelial cytoplasm (82). miR-155 ablation slowed the progression of central cord edema and reduced the incidence of paralysis. miR-155 was found to negatively regulate Mfsd2a *in vitro* (51). Li et al. reported that pretreatment with miR-320a mimic by intrathecal infusion attenuated IRI-induced lower-limb motor function deficits and BSCB dysfunction in SCIRI rats through downregulating the expression of AQP1 (59).

### Long Non-Coding RNAs in Spinal Cord Ischemia–Reperfusion Injury

Recent evidence has indicated that many lncRNAs are differentially expressed in SCIRI and may act as a significant part of the pathophysiology of SCIRI. Zhou et al. analyzed the expression pattern of lncRNAs in the SCIRI rat model using high-throughput RNA sequencing, suggesting that 6,707 mRNAs and 1,455 lncRNAs were differentially expressed, including 761 lncRNAs with increased expression and 694 with decreased expression (83). Furthermore, another study profiled the expression pattern of lncRNAs using a similar method and indicated that 7,980 lncRNAs (234 upregulated and 7,746 downregulated) and 3,428 mRNAs were differentially expressed (84). A bioinformatics analysis showed that those differentially expressed lncRNAs and mRNAs were associated with many biological pathways and processes, which might be crucial to provide new insight into the identification of potential therapeutic targets for SCIRI (83).

#### lncRNA CasC7

lncRNA CasC7 is about 9.3 kb in size, whose full name is cancer susceptibility candidate 7 (85). van Heesch and his team revealed that CasC7 exists in the nucleus and cytoplasm. When CasC7 exists in the cytoplasm, it will react with the large cytoplasmic polyribosome complex and participate in translation regulation (86).

It has been demonstrated that hydrogen sulfide (NaSH) preprocessing can decrease the spinal cord infarct zone in a SCIRI rat model and reduce the apoptosis rate in the SH5Y-SY cells treated with OGD/R. CasC7 expression was diminished and miR-30c was enhanced *in vitro* and *in vivo*, respectively. NaSH preprocessing can augment the level of CasC7 and inhibit the expression of miR-30c in SH5Y-SY cells treated with OGD/R. Furthermore, knockdown of CasC7 results in increased expression of Mir-30c and promotes apoptosis in this cell model (87).

Therefore, lncRNA CasC7 could upregulate the level of autophagy, protect against cell apoptosis and inflammation *via* inhibiting miR-30c, and increase SIRT1 expression *in vivo* and *in vitro* (44, 45, 87).

#### lncRNA MALAT1

MALAT1, which exists in various species, is the first lncRNA identified to promote the proliferation and metastasis of various cancers through alternative splicing and gene expression (88–90). MALAT1 is an abundant, quite conservative, and stable lncRNA (~7 kb) (91). MALAT1 is expressed in the skeletal muscle, vascular endothelial cells, and cardiomyocytes, which can be regarded to be involved in pathological myogenesis and angiogenesis (92–95).

Qiao et al. confirmed that MALAT1 exerted a neuroprotective effect in neurocyte lines under hypoxic conditions and the rat model of SCIRI by regulating miR-204. *In vivo* and *in vitro* experiments indicated that the expression of MALAT1 and Bcl-2 was reduced, while the expression of miR-204 was enhanced. Furthermore, overexpression of MALAT1 induced anti-apoptosis, and knockdown induced pro-apoptosis. However, the anti-apoptosis and pro-apoptosis effects can be reversed by synchronous overexpression and knockdown of miR-204, respectively. Moreover, SCIRI rats treated with MALAT1 exhibited higher levels of Bcl-2 expression, lower miR-204 expression, and lower Motor Deficit Index scores (96).

Therefore, overexpression of MALAT1 could decrease the expressions of miR-204, increase the expressions of Bcl-2, Beclin-1, the LC3-II/ LC3-I ratio, and RAP2C to attenuate apoptosis in the cells treated with hypoxia, and exert spinal cord protection against IRI (53–55, 96).

#### lncRNA TUG1

TUG1 is firstly identified in a genomic screen, which takes part in the development of the retina. In response to taurine, developing retinal cells can enhance the expression of TUG1 (97). The abnormal expression of TUG1 is strongly associated with different kinds of tumors (98, 99), myocardial ischemia (100), and neurodegenerative diseases (101).

The TLR4-mediated spinal microglial activation and NF-κB/IL-1β signaling pathway were involved in SCIRI, which initiated neuro-inflammation and neuro-apoptosis. Inhibiting TLR4 reduced the release of inflammatory cytokines after SCIRI (69). TLR4 interactor with leucine-rich repeats (TRIL), a receptor accessory protein of TLR4, significantly regulated the activation of TLR4 and the release of its downstream inflammatory cytokine IL-1b (102).

The mRNA levels of TUG1 and the expression of NF-κB/IL-1b signaling pathway were upregulated after SCIRI in rats. Activated microglia aggravated the neurological impairment and BSCB leakage after IRI. However, the knockdown of TUG1 can inhibit the inflammatory damage of IRI through decreasing the TRIL expression. Contrarily, the overexpression of TRIL can reverse the restraint of inflammatory effect. This study suggested that the regulation of TUG1 and TRIL may be a promising treatment strategy for SCIRI (103).

## Conclusion and Perspectives

### Conclusion

Despite the dramatic progress in surgical techniques and perioperative strategies of thoracoabdominal aortic aneurysm repair, the prevention of SCIRI remains a challenge for cardiovascular surgeons. An increasing number of studies indicated that ncRNAs play critical parts in neurological dysfunction after SCIRI and may pave the way to develop more novel molecular therapeutic options.

This review article is the first time to assemble the knowledge of ncRNAs regulating apoptosis, inflammation, autophagy, oxidative stress, or other processes to attenuate SCIRI. In fact, numerous research focused on the relationship between miRNAs and SCIRI in the last decades, while the functions of other ncRNAs in SCIRI need to be fully investigated in the future. lncRNAs, the emerging star ncRNAs, should become the focus of research in the field of SCIRI for the next decade.

### Perspectives

The translation of ncRNA research into clinical practice is a fascinating step. Even though miRNAs have “off-target” effects, they are ideal candidates for pre-clinical experimental research being highly conserved among species. Contrarily, in the ncRNA field, lncRNA, and circRNA are relatively new players. The poor conservation of nucleotide sequence between species and the presence of several transcripts are two major obstacles for scientists to clarify the specific signaling mechanism of lncRNA. With the ability to resist RNA exonucleases, circRNAs have great potential to become a biomarker for disease diagnosis.

At present, the ncRNA-based therapy aims to restore dysregulated expression levels of ncRNA to their basic level. Animal model experiments *in vivo* revealed that an intrathecal injection of mimic and exosome can effectively upregulate ncRNA. siRNA, shRNA, oligonucleotide, and inhibitor can downregulate ncRNA under the same operation. The exciting possibilities and enthusiasm of regulating ncRNAs to intervene in an SCIRI animal model can be tempered by some harsh realities. These therapeutic strategies are still far from our clinical setting. The disadvantages associated with regulating ncRNAs to treat SCIRI can be listed as follows: First, modifying cellular processes and molecular pathways to prevent SCIRI may not be as simple as expected around the bedside. Inferences drawn from experimental animal models may not successfully carry over to human systems. Second, modulating the expression of miRNAs may have undesirable side effects and unexpected consequences, which may have not been found in a short-term animal model. The regulation of ncRNA can promote a protumor environment (104–108).

Even though an increasing number of research results emphasized the promising therapeutic effects of ncRNAs, the wide clinic application of ncRNA-targeting molecules in SCIRI still has a long way to go.

## Author Contributions

XL, PZhou, SZ, and PZhu contributed to the conception and design of this review. XL, JL, and JY searched the literature and wrote the manuscript. XL, HQ, and XZ created the figure and table, and participated in drafting the manuscript. PZhou, SZ, and PZhu revised the manuscript. All the authors read and approved the final manuscript.

## Funding

This research was supported by grants from the National Natural Science Foundation of China (82170274 and 82100410), Guangzhou Science and Technology Planning Project (Grant No. 201804010067 and 2017ZC0064), and the President Foundation of Nanfang Hospital, Southern Medical University (Grant No. 2019c030 and 2017B022).

## Conflict of Interest

The authors declare that the research was conducted in the absence of any commercial or financial relationships that could be construed as a potential conflict of interest.

## Publisher's Note

All claims expressed in this article are solely those of the authors and do not necessarily represent those of their affiliated organizations, or those of the publisher, the editors and the reviewers. Any product that may be evaluated in this article, or claim that may be made by its manufacturer, is not guaranteed or endorsed by the publisher.

## Abbreviations

12/15-LOX: 12/15-lipoxygenase
3-MA: 3-methyladenine
3′-UTR: 3′-untranslated region
Bcl2: B-cell lymphoma-2
BSCB: blood–spinal cord barrier
circRNAs: circular RNAs
CSF: cerebrospinal fluid
dEX: dexmedetomidine
ECE1: endothelin-converting enzyme 1
FosDT: Fos downstream transcript
GLT-1: glutamate transporter-1
HMGB1: high-mobility group box-1
H_2_S: hydrogen sulfide
iASPP: inhibitory member of the apoptosis-stimulating proteins of p53 family
IPC: ischemic preconditioning
IRI: ischemia–reperfusion injury
lncRNAs: long non-coding RNAs
lncRNA CasC7: long non-coding RNA cancer susceptibility candidate 7
MALAT1: metastasis-associated lung adenocarcinoma transcript 1
MCAO: middle cerebral artery occlusion
Mfsd2a: major facilitator superfamily domain-containing 2a
miRNAs: microRNAs
mRNA: messenger RNA
NaSH: hydrogen sulfide
ncRNAs: non-coding RNAs
NOX: NADPH oxidase
OGD: oxygen-glucose deprivation
OGD/R: oxygen-glucose deprivation/reoxygenation
piRNAs: piwi-interacting RNAs
ROS: reactive oxygen species
rRNAs: ribosomal RNAs
SCIRI: spinal cord ischemia–reperfusion injury
siRNAs: short interfering RNAs
SIRT1: sirtuin 1
SNHG14: small nucleolar RNA host gene 14
TIMP3: tissue inhibitor of metalloproteinases-3
TLR3: Toll-like receptor-3
TLR4: Toll-like receptor-4
TNF: tumor necrosis factor
TNF-α: tumor necrosis factor-α
TRIL: TLR4 interactor with leucine-rich repeats
tRNAs: transfer RNAs
TUG1: taurine-upregulated gene 1
VEGF: vascular endothelial growth factor.
